# Sacrificial adhesive bonding: a powerful method for fabrication of glass microchips

**DOI:** 10.1038/srep13276

**Published:** 2015-08-21

**Authors:** Renato S. Lima, Paulo A. G. C. Leão, Maria H. O. Piazzetta, Alessandra M. Monteiro, Leandro Y. Shiroma, Angelo L. Gobbi, Emanuel Carrilho

**Affiliations:** 1 Laboratório de Microfabricação, Laboratório Nacional de Nanotecnologia, Centro Nacional de Pesquisa em Energia e Materiais, Campinas, São Paulo 13083-970, Brazil; 2 Instituto de Química, Universidade Estadual de Campinas, Campinas, São Paulo 13083-970, Brazil; 3 Instituto de Química de São Carlos, Universidade de São Paulo, São Carlos, São Paulo 13566-590, Brazil; 4 Instituto Nacional de Ciência e Tecnologia em Bioanalítica, Campinas, São Paulo 13083-970, Brazil

## Abstract

A new protocol for fabrication of glass microchips is addressed in this research paper. Initially, the method involves the use of an uncured SU-8 intermediate to seal two glass slides irreversibly as in conventional adhesive bonding-based approaches. Subsequently, an additional step removes the adhesive layer from the channels. This step relies on a selective development to remove the SU-8 only inside the microchannel, generating glass-like surface properties as demonstrated by specific tests. Named sacrificial adhesive layer (SAB), the protocol meets the requirements of an ideal microfabrication technique such as throughput, relatively low cost, feasibility for ultra large-scale integration (ULSI), and high adhesion strength, supporting pressures on the order of 5 MPa. Furthermore, SAB eliminates the use of high temperature, pressure, or potential, enabling the deposition of thin films for electrical or electrochemical experiments. Finally, the SAB protocol is an improvement on SU-8-based bondings described in the literature. Aspects such as substrate/resist adherence, formation of bubbles, and thermal stress were effectively solved by using simple and inexpensive alternatives.

In microfluidics, the effect of the material on the microchannel surface properties is amplified because of the large surface-to-volume ratio. As a consequence, the substrate dominates the functions of the microfluidic chips^1^. In general, glass is the best material for applications in microfluidics, including as additive steps and subtractive etchings to fabricate empty cavities (such as reservoirs, chambers, and microchannels) and positive units (as electrodes, monoliths, pumps, and valves)^2^. The thermal expansion coefficients (**α**) for glass, metals, and semiconductors are in the same order of magnitude. This fact avoids thermal stress and consequent cracking or shrinkage of metals and semiconductors deposited on glass during electrical or electrochemical experiments. Furthermore, the glass surface shows satisfactory electroosmotic mobility (**μ**_**EO**_) and thermal conductivity (**κ**, 1.5 W mK^−1^) for employment in electroosmotic pumping and capillary electrophoresis (CE) ensuring fast and high-resolution separations^3^. Other advantages of glass are: (1) optical transparency, (2) chemical inertia, (3) solvent resistance, (4) thermal stability, (5) robust fabrication, and (6) nano-scale adequacy, allowing us to encode nanostructures by using femtosecond laser writing^4^ or high-resolution lithography^5^. Meanwhile, the high complexity and cost of microfabrication (especially because the step of bonding that usually requires cleanroom, harsh temperature and pressure, and dedicated equipment), the difficult integration of functional components for the development of micro total analysis systems (μTAS), lab-on-a-chip (LOC), microelectromechanical platforms, and poor gas permeability (not viable for cell culture assays) limit a broader use of glass chips^1^.

In response to the drawbacks related to the microfabrication technology of glass chips, the field of microfluidics emerged just after the advent of poly(dimethylsiloxane) (PDMS) at the end of the 1990s^6^. Besides its simple soft lithography-based fabrication, the PDMS elastomer is adequate for the integration of functional elements^7^ and cell culture assays^8^,^9^. Conversely, PDMS presents important downsides, including: (1) permeability (it may spoil the results of vapour loss or pH change), (2) elasticity (the channel may deform depending on applied pressure generating, for example, unstable mass transport), (3) contamination of the sample with unreacted oligomers, (4) absorption of small molecules, (5) biomolecule adsorption, and (6) swelling in some organic solvents^10^,^11^,^12^,^13^. Thus, PDMS is restricted to analyses in aqueous media. Recently, Mays *et al.* reported a polymer that resisted the phenomenon of swelling in organic medium, namely poly(vinylmethylsiloxane)^14^. Furthermore, oxygen plasma-assisted irreversible bonding for PDMS microchips is cumbersome despite its simple procedure^15^,^16^,^17^. This downside is relative to the instability of the oxidized surfaces in air once the reactive silanol groups remain for only up to 10 min. It hampers the application of this bonding in ultra large-scale integration (ULSI) processes. Furthermore, functionalization procedures are not possible before the bonding process owing to the harsh conditions of the plasma. Such conditions can damage functional groups at the surfaces like organic chemical receptors. *In situ* modifications, in turn, are difficult owing to the inert and hydrophobic nature of native PDMS^18^. Furthermore there is a simple way to obtain hydrophilic PDMS surfaces, which is degassing under vacuum^19^. Lastly, PDMS is neither suitable for deposition of metals and semiconductors nor for electrophoresis applications. The thermal expansion coefficient of PDMS is typically one order of magnitude higher than the values for metals and silicon-based substrates. This fact may generate thermal stress, cracking, shrinkage, or even delamination of polymer structures over such surfaces. Another downside is the low adhesion between metals and PDMS^20^. Its limitations in electrophoresis arise from poor **μ**_**EO**_/**κ**^21^. Other compounds for specific situations include glass-elastomer hybrids, thermosets, thermoplastics, hydrogels, and paper. Recently, Ren *et al.* listed several substrates, which could be used depending on the final assay purpose^1^.

Considering the notable features of the glass for microfluidics and drawbacks related to polymer substrate—especially PDMS—as previously stated, the development of methods to reduce the costs and complexity of the bonding step for glass microchips is a current and active research field. The conventional method used to seal these microdevices is thermal bonding^22^. It is a direct approach based on condensation reactions (reaction (1) on “Adhesion strength” section) involving silanol groups (–SiOH) at high temperatures that are usually between 450 and 650 °C for all-glass surfaces^23^. Thermal bonding is applied to microchips of silicon, glass, and quartz and it generates appreciable interface energies (adhesion strength). Nonetheless, it is essential that the substrate surfaces are flat (root mean square, RMS, on the order of nm) and very clean. Therefore, the bonding step should be conducted in a clean room with heavy investment in its installation and maintenance. In addition, the harsh conditions of temperature may generate thermal stress, damaging the deposited thin films for electrical or electrochemical analyses^3^.

Herein, we present a new bonding method for fabricating glass microchips, named sacrificial adhesive bonding (SAB). The procedure is simple, fast, and compatible with ULSI processes and thin film integration. First, it consists of using an intermediate layer to seal glass slides to one another, as in conventional adhesive bonding. However, this intermediate layer is selectively developed (and removed) to create microfluidic channels with glass-like surface properties. In this procedure, a specific developer flows with the aid of a hydrodynamic pumping system removing the intermediate just inside the microfluidic channel. The main steps of the microfabrication in SAB are depicted in Fig. 1. SU-8 resist was used as sacrificial adhesive. Such a thermoset is a fundamental element of pattern transfer processes obtained by photolithography. The advantages of this epoxy-based negative photoresist are: (1) thermomechanical stability, (2) chemical inertia (resistance to organic media and alkaline or acid solutions at high temperature), (3) transparency (near UV and visible radiation), (4) sensitivity, selectivity, and contrast to UV, (5) photolithography resolution, (6) biocompatibility, (7) vertical sidewall profile (desired for lift-off), (8) wide range of thickness (1 to approximately 500 μm) achieved by spinning in a single run^3^, and (9) electrophoretic performance similar to that of glass^24^. Additionally, uncross-linked SU-8 is used as glue in adhesive bonding processes. All-SU-8 microdevices are reported in the literature as well. The latter require a multilevel photolithography for fabrication that defines the bottom, sidewall, and top surfaces in SU-8^25^.

The current paper reports the SAB fabrication steps as well as investigations into the repeatability of the glass etching step, the decrease in bubbles and thermal stress, the uniformity of the SU-8 film, the transversal section of the microchannel, the adhesion strength between the stacked flats, and the effect of the remaining SU-8 on the properties of microchannel surface. Finally, we performed a comparative study involving the developed bonding and some techniques for glass chips from the literature.

## The approach

### General Considerations

**Figure 1:**
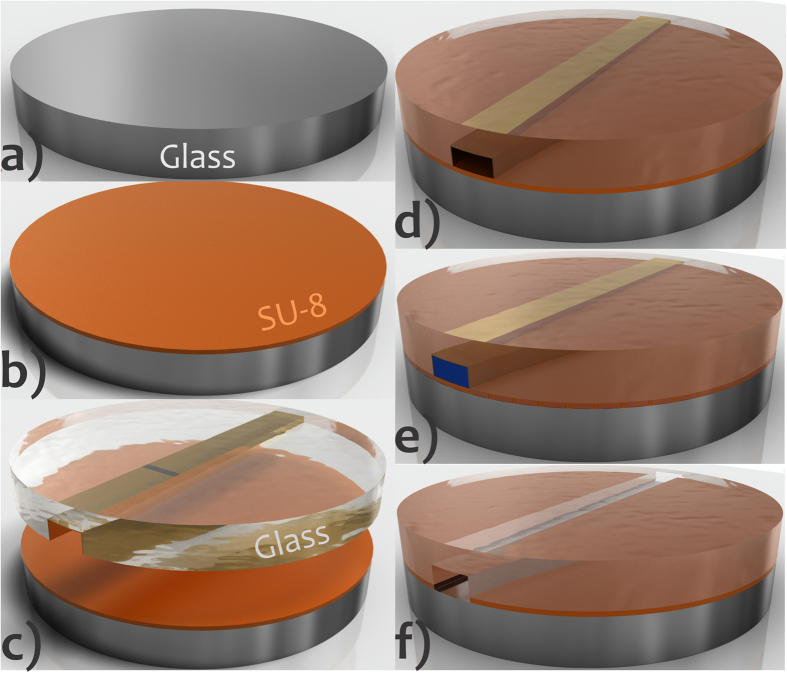
Microfabrication steps for SAB. Glass slide (**a**), deposition of SU-8 over this wafer and then pre-bake (**b**), preliminary bonding against the glass substrate with the walls of the microchannel coated with a thin film of Al (golden in the drawings) (**c**), cure of the resist just around the microchannel after UV exposure and PEB (resist under the channel is not cured because it is protected from UV by thin film of Al that is deposited only inside the microchannel; see Fig. 4) (**d**), removal of the uncured SU-8 and, next, of the Al film by pumping specific solvents (blue in the drawing) (**e**), and final chip showing residual SU-8 in the bottom of the sidewalls (Fig. 6) (**f**). Features not drawn to scale. In addition, the sidewalls in wet-etched glass are commonly not vertical but rounded.

The main steps of the SAB (Fig. 1) include: (1) deposition of adhesive on a glass flat slide, (2) preliminary bonding against the substrate integrating microchannels coated with thin-film, (3) cure of the resist between the glass surfaces around the channel, and (4) development of the uncured adhesive resist.

In general, adhesive bonding is attained only after curing (polymerization) of the adhesive intermediate^26^. In this case, highly aggressive solvents would be required for the development step in SAB, damaging the bonding itself and any functional components integrated in the microfluidic device. The selection of SU-8 like adhesive provided preliminary bondings containing high adhesion strength when only SU-8 monomers (uncured resist) were applied. It allowed us to use mild solvent (SU-8 developer) for the selective removal of adhesive. The chemical inertia is another advantage of SU-8. It is important because residual layers of this resist remain at the microchannel sidewalls after the SAB process. Such layers may interact with the sample in analytical applications, generating unpredictable results.

Besides the need of using uncured resist for step of adhesive removal as discussed above, other hurdle is the selective development of the adhesive only inside the channel. A simple alternative to this step concerns the pumping of the developer at a specific time and flow rate. Nevertheless, the rate of development for SU-8 is very high as some experiments demonstrated. Thus, an excessive removal of the adhesive layer in regions beyond the microchannel was observed even at a very low time (10 s) and developer flow rate (5 μL min^−1^) (described below). Herein, one option would be to dilute the developer with a suitable solvent, but such a procedure is not suitable for ULSI processes because a new calibration for development time and flow rate would be necessary for different adhesive thicknesses. Hence, a method based on curing the SU-8 resist only around the microchannels before preliminary bonding was created. This process avoided the excessive removal of adhesive and new calibrations depending on thicknesses of SU-8.

**Figure 2:**
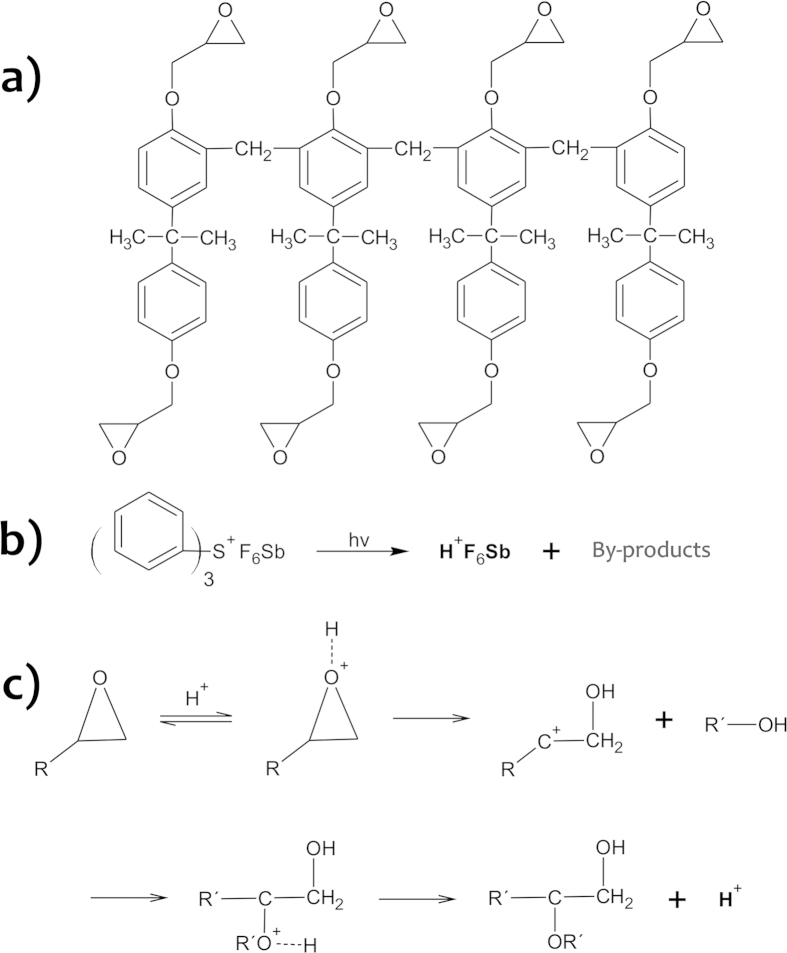
Chemical structures and reactions in SAB. SU-8 structure. (**a**), activation of the triphenyl-sulfonium herafluoroantimonate (initiator of the cure) at UV (**b**), and (**c**) polymerization of the monomers in PEB.

Cure of the SU-8 requires two sequential steps: (1) UV exposure at 365 nm for formation of the photoactivator and (2) post-exposure bake (PEB) to allow the polymerization of SU-8 monomers^26^,^27^. The chemical structures and reactions involved in this process are depicted in Fig. 2^3^,^21^. Condensation reactions between glass and resist surfaces provide an irreversible bonding. The selective development of the SU-8 monomers underneath the microchannel requires that such regions be protected from UV radiation. In this situation, there is not the cure of resist inside the microchannel because the polymerization initiator is not formed. For this process, we used a photolithographic negative mask that relies on an Al opaque film deposited inside the microchannel. This thin film of Al acted as an absorption standard (a template) during UV exposure, protecting the SU-8 under channel from radiation, whereas all of the other regions were exposed, and, then, later cured. Accordingly the development step in SAB can be conducted for long time and high flow rates regardless of the adhesive thickness as the side layers are already cured during such a procedure.

### Microfabrication

Microfabrication relied on standard UV photolithography for the pattern transfer processes, including (1) deposition of thin films by spinning and sputtering and (2) wet etching.

#### Carving of the microchannels and ATZs

**Figure 3:**
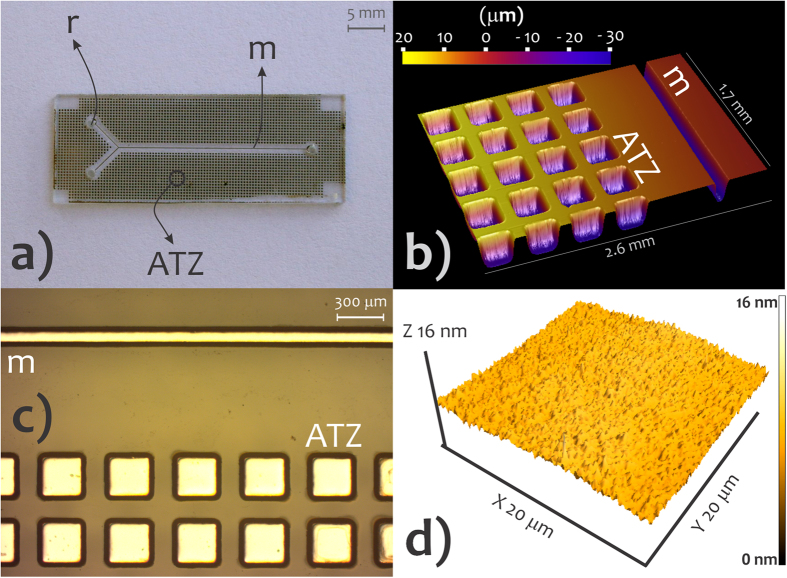
Microchannels, ATZs, and SU-8 surface morphology. Photo of the substrate incorporating channel and ATZs (**a**), profilometry image of microchannel and ATZs (**b**), photo by digital microscope showing the Al film-coated cavities (**c**), and AFM image of the SU-8 surface (**d**). m, microchannel; ATZ, air-trapping zone, and r, reservoir.

Cavities around the microchannel called air-trapping zones (ATZs) were engraved on substrate at the same time as the etching of the microchannels These structures are illustrated in Fig. 3. That being so, the etching mask covered both the regions for microchannel and ATZ. The latter aims to retain the air bubbles created during the preliminary bonding in the interface of glass slides^28^. Thus, ATZs improve adhesion strength and bonding quality (measured by reducing the defect rate).

Standard photolithography transferred the pattern by utilizing a photolitho-based dark field (positive) mask, AZ 4620 positive resist, photoaligner (Karl Suss America Inc. MJB 3 UV 200, Waterbury, VT), thin film deposition by spinning (Headway Research Inc. EC101DT, Garland, TX), and corrosion by wet etching. The procedure for etching was performed sequentially with the following solutions: (1) 396 mL H_2_O, 54 mL Hydrofluoric acid (HF), and 270 g NH_4_F for 14 min; (2) 150 mL H_2_O, 150 mL hydrochloric acid (HCl), and 150 mL HF for 3 min; and (3) HCl for 1 min. Then, substrates were rinsed in excess acetone with the aid of ultrasound, washed with deionized water, and dried in N_2_. The channels were Y-shaped in geometry (Fig. 3). Reservoirs were fabricated with a diamond drill. Repeatability of the glass etching was studied by measuring width and depth in 11 slides through profilometry (Veeco Dektak 2210, Branson, MO) for five distinct points on each slide.

#### Fabrication of the mask for SU-8 selective cure

**Figure 4:**
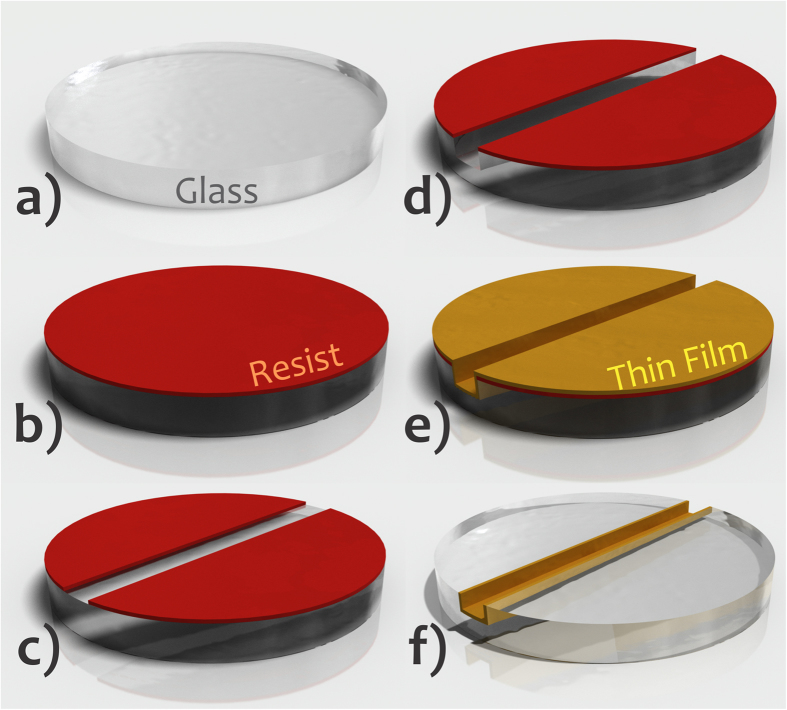
Fabrication of the mask for SU-8 selective polymerization by depositing Al thin film only inside the microchannel. Glass flat slide (**a**), deposition of positive resist over this slide and then pre-bake (**b**), UV exposure, development producing the mask for microchannel pattern transfer, and hard bake (**c**), glass etching (**d**), deposition of opaque thin film by sputtering over all of the slide (**e**), and lift-off with the thin film only inside the etched cavity (**f**). Features not drawn to scale.

As explained earlier in “General Considerations” section, the mask for selective cure of adhesive resist was based on the deposition of a film of Al inside the microchannel. Al is ideal because it is cheaper than other metals like Au and is easily removed in HF or alkaline diluted solutions—a required step after SU-8 development. Vapour phase deposition methods are potential alternatives taking into account their high production capacity and film uniformity. The protocol applied in this specific process is depicted in Fig. 4. The resist for engraving the microchannel and the ATZs was retained after etching of the glass slides. Next, Al thin film was deposited *via* sputtering (Oerlikon Balzers BA510, Schaumburg, IL). Lastly, acetone excess lifted off the etching mask for 2 min. As a consequence, a selective coating of the cavities by Al was attained. The developed method avoids both alignment and further photolithography steps applied in standard processes for pattern transfer of the microchannel and ATZs by wet etching.

#### Bonding

The protocol was the same as that previously reported for hybrid glass/SU-8 chips—without the selective development of SU-8^26^. It was an improvement compared with the approaches usually applied for SU-8^25^. Bake times were decreased and additional steps to raise the glass/SU-8 adhesion or progressive temperature ramps were not necessary. In addition, ATZ reduced the formation of void areas and eliminated thermal stress.

The main steps of bonding are: (1) spin coating SU-8 onto the glass slide, (2) preliminary bonding between this slide and substrate with microchannel, ATZs, thin film of Al as absorption standard, and reservoirs, (3) SU-8 polymerization just around the microfluidic channel, (4) resist development and Al removal by pumping specific solvents inside the microchip, and (5) hard bake (see Fig. 1 for a more didactic explanation).

Bonding was achieved using SU-8 5 (52% m/m in gamma-butyrolactone, GBL) as adhesive layer in a temperature above resist ***T***_***g***_ (glass-transition temperature) and native SU-8. Initially, the slides were chemically cleaned through immersion in piranha solution (H_2_SO_4_/H_2_O_2_ 2:1, v/v) for 10 min to remove organic impurities. Afterwards, the slides were dehydrated at 120 °C on a hot plate for 30 min. It improved the adhesion of the glass/SU-8 by decreasing the surface energy. Moreover, the use of reactive silicones like hexamethyldisilazane (HMDS) was not required^3^. SU-8 was coated on glass chips by spinning; deposition conditions were investigated as discussed below to increase the uniformity of the film. As the dehydration step, the usage of thin and low-viscosity films ensured a good adhesion of the SU-8 film to glass slides^29^. Ways to improve the adhesion of the film include: (1) substrate oxidation in O_2_ plasma^30^, (2) coating of the substrate with reactive silicone primers^3^ or ultra-thin Ti adhesion layers^31^, and (3) doping of resist with hydrophilic species^32^,^33^. Soft bake, which is intended to remove solvent, occurred in a two-step process: the SU-8 was soft baked at 65 °C for 1 min to decrease thermal stress and then at 95 °C for 5.0 min. After 2.5 min of soft bake, the SU-8-coated glass was brought into contact with substrate under the application of a constant weight of 200 g. This step was conducted until the end of soft bake step, thus during 2.5 min at 95 °C. The hot plates were horizontally leveled since the gravity can affect the flatness of resist thin films. It is important also to highlight that progressive temperature ramps were not employed. Next, the cure of the resist just around the microchannel and ATZs (because the presence of the Al inside these cavities precluded polymerization) was performed as usual^25^. Exposing the microchip at 365 nm UV activates the photoinitiator and PEB ensures the polymerization reactions as shown in Fig. 2. Experimentally, the microdevices were exposed at 365 nm UV for 1 min with an exposure dosage of 600 mJ cm^−2^. The exposure was conducted from the top of substrate to protect the resist under microchannels of UV radiation by means of the Al mask. Hence, the SU-8 in this region was not cured allowing its posterior removal by using a specific developer. Afterwards, the cure of SU-8 was performed during the PEB at 65 °C for 1 min and then at 95 °C for 1.0 min as well. The exposure extension is critical because short times produce less dense cross-linked polymers. Therefore, the solvent molecules diffuse into SU-8 bulk swelling the film during its development^34^. PEB raises adhesion, reduces scumming, and improves the contrast and profile of the resist^3^. Recently, Mitri *et al.*^35^ described another way to cure SU-8. They eliminated the PEB by exposure at 254 nm UV. It was enough to promote the epoxy ring opening, thus allowing polymerization.

To execute the development of the SU-8 and removal of the Al film, the microchip was previously connected to syringe pump by means of stainless steel needles, which were fixed by epoxy glue on the reservoirs in substrate. Silicone tubes ensured the contact between syringe and needles. The protocols of SU-8 development and Al removal consisted of flowing propylene glycol monomethyl ether acetate (PGMEA, 40 s at 20 μL min^−1^) and then 10% v/v HF in water (2 min at 50 μL min^−1^) through a syringe pump (New Era Pump Systems Inc. NE-300, Wantagh, NY) inside the microchannel, respectively. Excessive removal of SU-8 through the sidewalls of the microchannel was not verified because their edges were already polymerized during this stage. Finally, the microfluidic channel was washed by pumping deionized water for 3 min at 50 μL min^−1^.

## Results and Discussions

### Etching repeatability

Glass etching for carving of the microfluidic channels showed satisfactory repeatability levels. Average global values (*n* = 55) were 29.8 ± 0.1 μm (depth) and 116.0 ± 2.0 μm (width). Relative standard deviations (RSD) for the same slide (intra-chip precision) exhibited average values of 0.1% and 2.3% for depth and width, respectively. In addition, the RSD were 0.2% (depth) and 2.9% (width) for different glass slides (inter-chip precision). As regards to ATZ cavities, their average dimensions for five slides (*n* = 25) were 28.7 ± 0.1 μm (depth) and 293.0 ± 2.9 μm (width).

### Formation of bubbles

**Figure 5:**
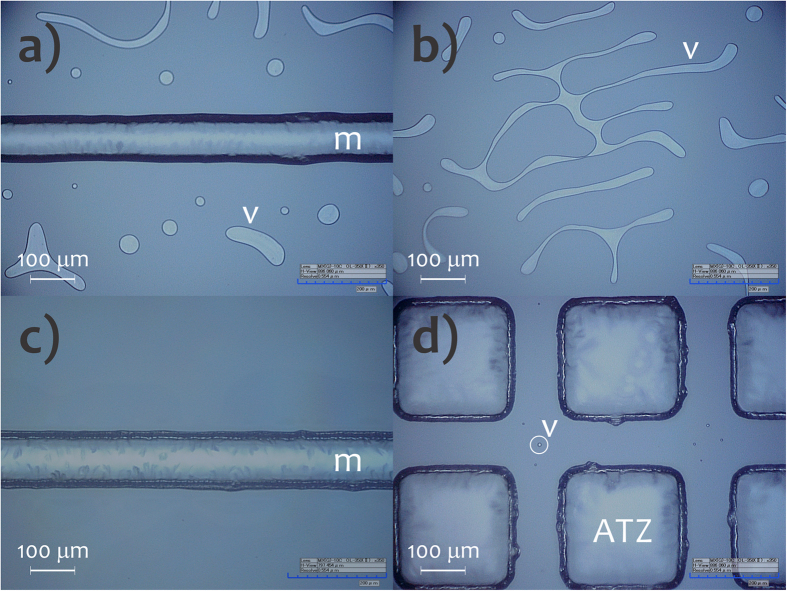
Optical microscopy images of the microchips without (**a**,**b**) and with (**c**,**d**) ATZs. m, microchannel and v, voids and air bubbles.

The ATZ cavities significantly decreased the formation of voided areas by retaining the air produced during the preliminary bonding, as illustrated in Fig. 5. We can verify that there was a very high incidence of bubbles when the ATZs were not used. Voided areas help to minimize the adhesion strength and can affect the dimensions and shapes of integrated empty spaces and positive features in the analytical microdevice. Furthermore, shrinkage or cracking of the SU-8 films was not verified. This fact indicates the effectiveness of the ATZ cavities in reduction of thermal stress by decreasing contact surface between SU-8 and glass. Some alternatives for reducing thermal stress further are: (1) low bake temperatures^36^,^37^, (2) doping of the resist based on nanoparticles of SiO_2_^38^, and (3) treatment in ultrasound^39^.

### Uniformity and roughness of the SU-8 film

SU-8 adhesive was deposited over the glass by spinning during 30 s. In general, it is the minimum spin time necessary to generate a uniform coating. This parameter is essential for bonding adhesion strength by determining the density of defects and, thus, the contact area between adhesive and substrate. The deposition was conducted using two procedures. First, the spin coating was based on a unique speed (3000 rpm as described in Methods section, process **A**). The other deposition mode was performed using two steps of velocity in order to improve the uniformity of SU-8 by decreasing edge beads and ridges (process **B**). These steps were: spread cycle (500 rpm for 5 s) enabling the spread of fluid on the entire substrate and, then, spin cycle (3000 rpm for 25 s) defining the final film thickness. At these speeds, initially the resist flows to the slide edges because centrifugal force. Next, the fluid is expelled from the edge after building up when surface tension is exceeded^3^.

A substantial improvement in uniformity was found when the SU-8 film was deposited using the two-step spin profile, process **B**. Average global thicknesses were 3.76 ± 0.41 μm and 4.35 ± 0.08 μm for **A** and **B**, respectively. The process **B** was, then, applied in SAB. Figure 3(d) depicts an AFM image of the SU-8 surface obtained by routine **B**. It was smooth with RMS of 0.59 nm.

### Cross-sectional analysis of the microchannel

**Figure 6:**
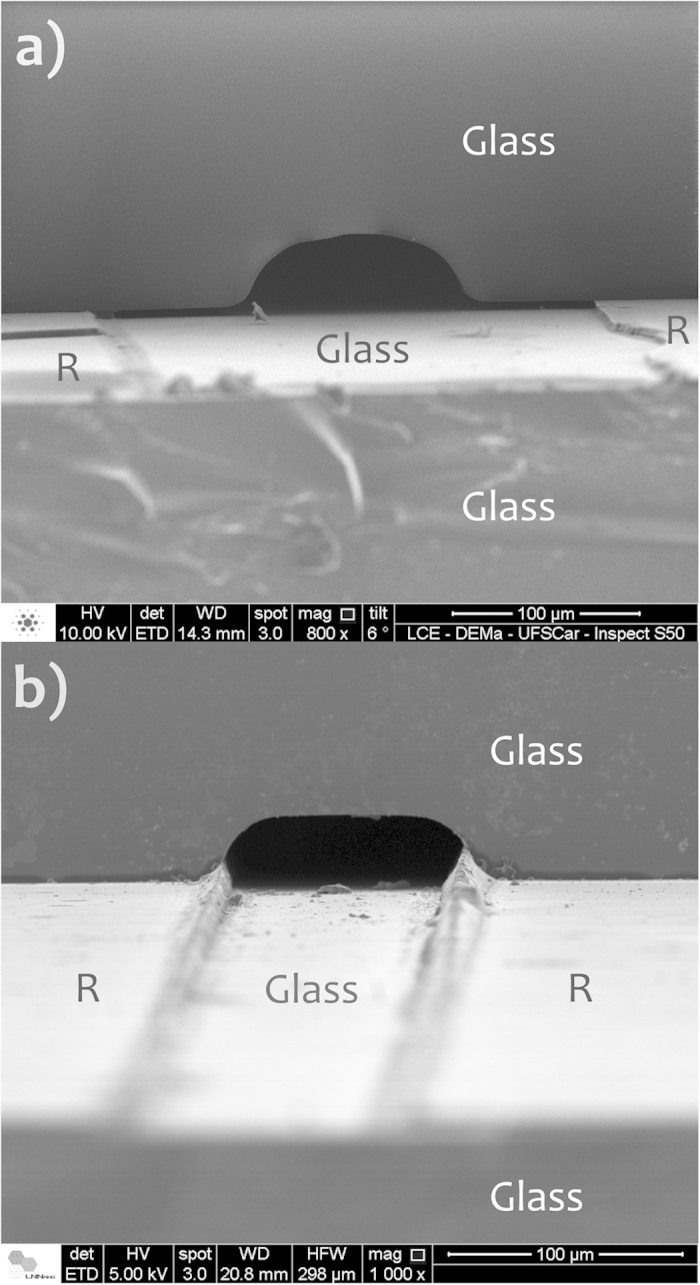
SEM-FEG micrographies of the cross-section of microchannels obtained by SAB for two methods of SU-8 development: prior to its cure (**a**) and after its cure outside the microchannel (**b**). R, SU-8.

Figure 6 shows micrographies of the cross-section of the bonded microchannel for the two methods of SU-8 film development in SAB discussed above. Figure 6(a) presents the excessive removal of adhesive when the development step is performed before its cure. In this case, PGMEA was pumped for 10 s at 5 μL min^−1^ for development of SU-8. Conversely, Fig. 6(b) illustrates the removal of the adhesive layer just under the microfluidic channel by using Al thin film as a mask. This mask allowed the resist removal after the selective cure around the microchannel during UV exposure. The procedure was conducted for 40 s at 20 μL min^−1^ of PGMEA.

The bonding temperature of 95 °C is greater than SU-8 *T*_*g*_ (64 °C)^40^. This temperature is important because it decreases the viscosity of the resist, bypassing voided areas which increase the contact surface and thus the adhesion strength during preliminary bonding. Conversely, very high values of pressure and temperature result in partial filling of the microchannel by the adhesive resist. This phenomenon arises from the low viscosity of the adhesive resist that flows inside the microfluidic channels due to pressure and capillary force^41^. The effects of filling are more pronounced when thicker films are used. For a 50 μm SU-8 layer, for example, the complete filling of channels with a depth less than 100 μm is verified at 68 °C^42^. Low temperature and pressure, in turn form voided areas, decreasing the adhesion strength and affecting the structure of units integrated in the microchip^28^. These parameters applied in SAB avoided the formation of bubbles. Conversely, a proportion of approximately 5% of the residual SU-8 adhesive was spread into microchannel edges, forming a semi-spherical cross-section as verified in Fig. 6(b)^26^. We believe this phenomenon is positive because the rounded sidewalls in etched amorphous glass (under etching) favour particulate retention in prolonged analyses or increase the band broadening in separation applications. Such residual SU-8 comes from the pressure and capillary forces during preliminary bonding that spread the resist inside the channel. Additionally, the polymerization of these residual parts is presumably caused by UV diffraction through the glass (Fig. 1(d)). It can generate the activation of the initiator (Fig. 2(c)) ensuring the cure of SU-8 in the microchannel edges during the PEB step.

### Adhesion strength

**Figure 7:**
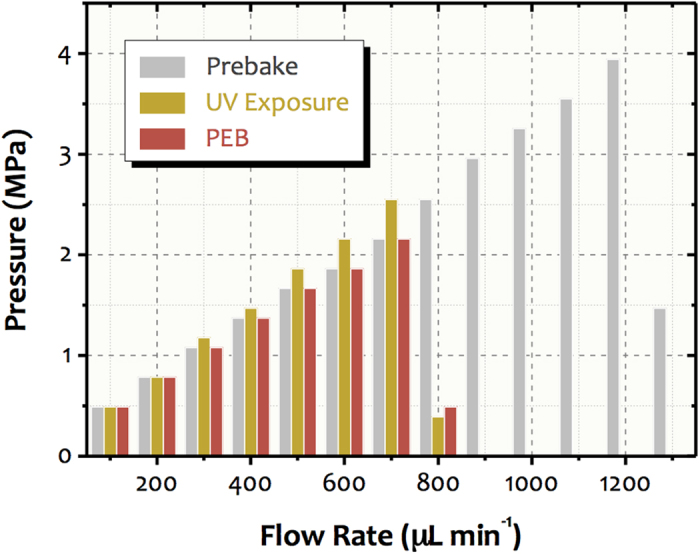
Testing the adhesion strength. This graphic shows the pressures inside the channel arising from flow rates applied by an HPLC pump. The chips were bonded with SU-8 adhesive in different processing stages: (1) post pre-bake (uncured, grey), (2) post-UV exposure (with produced cure initiator but uncured, yellow), and (3) post PEB (cured, red).

The results for the leakage tests and adhesion strength are presented in Fig. 7. Microfluidic channels did not show any leakage for any of the applied flow rates. However, the connections between the pump and the microchips leaked over 800 μL min^−1^ for both SU-8 adhesives obtained post UV exposure and PEB and over 1,200 μL min^−1^ for the resist attained post pre-bake. Consequently, sudden reductions in pressure were observed. Taking up the tested flow rates (previously to the leaks in connections), the microchips endured pressures of up to 3.9 MPa. In addition, we can state that the preliminary bonding presented an adhesion strength high enough to withstand the pressures applied during the selective development step (flow rate lower than 100 μL min^−1^).

Regarding the tensile pull tests, all samples fractured over a pressure of 5.28 ± 1.04 MPa (*n* = 6). Moreover, the long-term performance of the bonding under high pressure was tested. The microchips (*n* = 3) endured a pressure of 2.00 MPa for 24 h without any fracture. The adhesion results demonstrated that the bonding has bursting pressures sufficient to withstand harsh flow rate conditions for long periods. In microfluidics, the flow rates are usually up to hundreds of μL min^−1^, producing pressure values on the order of 1 MPa only. According to investigations reported in the literature, SU-8-based bonding shows bursting pressures between 4 and 45 MPa^43^,^44^,^45^. These values are much larger than those attained when the PDMS is employed as adhesive layer. Using oxidative pretreatment by inductively coupled high-density plasma, we found that the maximum adhesion strengths for PDMS-PDMS and glass-PDMS were nearly 0.4 and 0.5 MPa, respectively^17^. For the glass-PDMS functionalization-assisted bondings, such force was only 0.6 MPa with grafted polymeric adhesive^18^.

The irreversible bonding between glass and cured SU-8 is owed to condensation reactions^22^ generating chemical adsorption by covalent bonds involving silanol groups as follows:


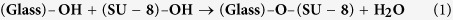


Another element that contributes to such bonding is the intrinsic adhesion. It is related to attractive intermolecular forces among the surfaces producing physical adsorption. This adhesion rises with the decrease in interfacial tension. For the glass and uncured SU-8, we believe that the condensation reactions also have some effect on irreversible bonding as well as intrinsic adhesion. In this case, the covalent bonds would be caused by the opening of the resist epoxy groups by the silanol groups present on the glass surface, thus allowing condensation reactions to occur. Although uncured SU-8 produced a satisfactory adhesion strength, its cure is essential for improvements in properties such as chemical inertia, thermal stability, and electroosmotic flow.

### Effect of the remaining SU-8 on channel property

The heat dissipation capacity depends on diverse factors such as (1) roughness, dimension, and composition of the microchannel wall and (2) temperature, pH, and solution ionic strength^46^. Because of the good etching precision when the microchannels were carved as described above, the results of thermal characterization can be attributed to the nature of the channel walls only.

**Figure 8:**
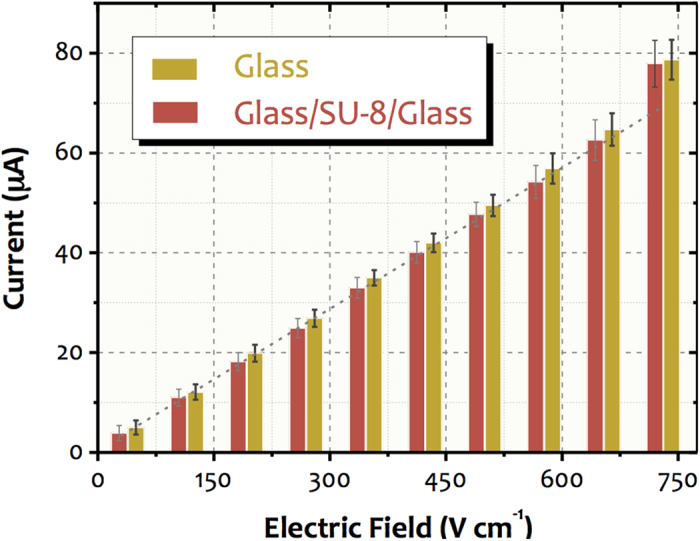
Effect of the remaining SU-8 layer on the surface properties of the microfluidic channel. The Ohm’s law plots were obtained by using 20 mmol L^−1^ phosphate at pH 8.0 for glass (yellow) and glass/SU-8/glass (red) microdevices. Each value represents a global average current which was achieved from four microchips (real-time monitoring for 2 min).

Under the same ionic strength conditions, the glass and SU-8 surfaces generate similar electroosmotic mobility^26^ and electrokinetic experiments were not necessary to study the effect of the SU-8 on the channel surface properties. Conversely, the thermal conductivity values for SU-8 and glass are 0.2 and 1.5 W mK^−1^, respectively. It yields a substantial difference in the heat dissipation capacity for glass and glass/SU-8 walls. Analyses with 20 mmol L^−1^ phosphate buffer at pH 8.0 showed some Joule heating with linearity deviation in the current/potential relationship at electric fields higher than 580 and 730 V cm^−1^ for microfluidic channels of glass/SU-8 and glass, respectively^21^. Under such conditions, chips obtained by SAB had similar thermal behaviour when compared to glass, as illustrated in the Ohm’s law plots of Fig. 8. In both cases, Joule heating was observed only at electric fields greater than 730 V cm^−1^. This data indicates that the residual layers of SU-8 after the SAB did not interfere on surface properties of the glass channel walls.

The medium employed to remove Al in SAB (10% v/v HF in water) also etches glass. Nevertheless, the similarity between the obtained currents for glass and glass/SU-8/glass (SAB) chips shows that the removal process of Al with such a solution did not interfere substantially on channel surface features. Presumably, this fact stems from the short exposition time and the laminar flow generated inside the microfluidic channels. The latter reduces the etching rate compared with the turbulence transport. Lastly, the deviations for diverse microchips indicated satisfactory repeatability within all the microfabrication processes.

### Comparative study

Alternatives for all-glass microchip bonding were developed because of the drawbacks of thermal bonding previously described. Generically, these methods consist of direct techniques at low temperature or even room temperature.

The protocols described in the literature for reducing temperature include (1) long periods of contact among the slides (150 °C)^47^, (2) surface activation in liquid (90^48^, 115^49^, and 65 °C^50^) and gas (70 °C^51^) phases, and (3) functionalization with silanol/amine groups (200 °C)^52^. Direct bondings of glass at room temperature are achieved by surface activation in plasma or in liquid phase. The processes applied in the first case are (1) argon atom fast beam^53^, (2) reactive ion etching (RIE) in plasmas of O_2_ radio frequency and N_2_ microwave^54^, and (3) RIE in O_2_ and CF_4_ plasma^55^. The activation in liquid phase^56^, in turn, is based on a laborious process that involves washing, immersion in acid solution for 8 to 12 h, exposure to tap water flow and demineralized water, and drying. Usually, the aforesaid methods aim to improve adhesion strength by increasing the density of silanol groups—reaction **(1)**. Table 1 lists some key features of these methods and compares to SAB.

Direct bonding at low temperatures decreases the risks of thermal stress and requires a cleanness level lower than that needed for conventional thermal techniques. Conversely, this method has several surface chemical treatment steps, which can take up to 24 h. Such a procedure is inadequate for the ULSI process. Methods at room temperature bypass thermal stress. The plasma activation-based technique has higher operation velocity. Nonetheless, it requires sophisticated, expensive, and dedicated instrumentation. Finally, activation in the liquid phase uses a very laborious and slow experimental protocol.

The SAB process proved to be a powerful approach to the fabrication of glass microfluidic devices. The protocol meets important requirements of an ideal method such as: (1) throughput (number of slides that can be transferred per hour), (2) relatively reduced cost, (3) compatibility with ULSI (surface oxidation in plasma or functionalization are not required), (4) high adhesion strength, holding-on pressures of at least 3.9 MPa, and (5) the properties of the microchannel are majorly governed by glass as indicated by the thermal dissipation test, keeping desirable features in terms, for example, of its application in electrokinetic assays. In addition, the SAB process (1) avoids the application of high temperature, pressure, or electric potential, enabling the deposition of thin films and eliminating thermal stress, (2) demands low levels of cleanness and flatness of the substrate (it tolerates micro-scale particles owing to the planarization by adhesive), thus bypassing the need for cleanrooms, and (3) does not create blocking of the microchannel by the adhesive layer. It is important to highlight also the advantages related to the use of the SU-8 as adhesive. Besides parameters such as thermal stability, chemical inertia, and optical transparence, this polymer does not show serious drawbacks as PDMS does, including non-specific adsorption and swelling in organic media. Moreover, the experimental protocol employed for the SAB process was improved in relation to the SU-8-based bondings described in the literature^25^. Aspects such as substrate/resist adherence, formation of bubbles between the slides, and thermal stress were effectively solved by using simple and inexpensive alternatives. The dehydration and PEB steps, as well as the use of a thin adhesive layer, ensured a good glass/SU-8 adherence. The ATZs, in turn, effectively reduced the formation of bubbles and void spaces, besides decreasing the thermal stress; cracking or shrinkage of the films deposited on the glass surface during the pre-bake, PEB, and post-bake steps were not observed. It is notable that for a high density of carved microstructures on glass, these own structures would act as air-trapping zones bypassing the use of ATZs in this case. Therefore, the trapping zones do not reduce the density of channel per unit area for array-based devices. Another positive feature of the SAB relates to the bonding of long (values of up to 65 mm were investigated) and wide (values of up to 140 μm were tested) microchannels without the formation of void areas or appreciable blocking of the channel as shown in Supplementary Information (Fig. S1). This drawback is an important limitation for most of the SU-8 adhesive-based bonding below^57^ or above the *T*_*g*_ of the resist^41^. The applied pressure and temperature generated a slight filling of the under-etched channels by the SU-8 layer, making the microchannel rounder and giving it a less characteristic shape. We believe such a phenomenon is satisfactory because it reduces the retention of particulate during use.

We believe the SAB represents a potential breakthrough in glass bonding technologies. The fabrication of the mask for selective development of SU-8 only inside the microchannel does not involve further photolithography steps in relation to the process required for carving microfluidic channels in glass by etching. Accordingly, the bonding needs only one step of photolithography to cure SU-8. In addition, this process does not need alignment, as shown in Fig. 4, a key factor for large-scale integration. The bonding step (deposition of SU-8, pre-bake, preliminary bonding, photolithography to cure the resist, and removal of the uncured SU-8 and Al) lasts approximately 40 min after the substrate once the channels and Al film have been prepared. Such a photomask involves only one additional step to those applied to fabricate the microchannels (UV photolithography, etching, and removal of resist). This additional step is the coating with Al. It lasts approximately 40 min at 10^−5^ mTorr in the sputter for deposition of hundreds of slides with 100 nm of Al as database of metal deposition in our group. Such a condition is amenable since the purity of the thin film of Al is not relevant in SAB, as it needs only to be opaque to protect SU-8 from UV light. Additionally, the coating of the microchannel with Al film can be performed by utilizing low-cost vapour phase deposition equipment. One example is sputters used to raise the contrast in microscopy images. Such a procedure is accessible to most research centers and it is ideal for ULSI. Additionally, the use of a physical mask during UV exposure to protect the SU-8 film is a potential approach for laboratories with less infrastructure. Photolitho-based masks are a cheap alternative^58^. In this case, the exposure is caused by shadow printing, not by contact printing^3^. Therefore, studies about the effect of light diffraction on resolution should be conducted. Finally, it is important to highlight that the application of the SAB process for channels presenting very small depths is limited by the adhesive thickness for two reasons. Initially, the intermediate film would interfere the channel surface properties for high ratio values between exposed areas of the adhesive and substrate. In addition, such channels could be clogged because of filling by the adhesive film during the preliminary bonding. For SU-8, a minimum thickness on the order of 1 μm is achieved by spinning^3^. Herein, channels with depth greater than micrometres could be bonded/presumably by using the SAB. Conversely, direct bonding is the most recommended way to bond nanofluidic devices^59^. Other limitations related to SAB include: functionalization processes before bonding are not possible and applications using fluorescence detection suffer with the auto-fluorescence phenomenon of SU-8. It decreases the sensitivity and detectability levels in the UV range^24^.

## Methods

### Chemicals

Soda-lime glass slides (25 × 45 × 1 mm) were supplied by Glass Técnica (São Paulo, Brazil). HF, ammonium fluoride (NH_4_F) and HCl were purchased from Synth (Diadema, Brazil). Sodium hydroxide (NaOH) was acquired from Sigma-Aldrich (St Louis, MO). SU-8 1000 photoresist, GBL, and PGMEA were purchased from MicroChem Corp. (Newton, MA). Monobasic sodium phosphate (NaH_2_PO_4_, used as phosphate buffer) was acquired from Mallinckrodt (Xalostoc, Mexico). Solutions were prepared in deionized water (Milli-Q, Millipore Corp., Bedford, MA) with resistivity no less than 18 MΩ cm.

### Formation of bubbles between the slides

Cavities around the microchannel were engraved on the substrate to retain the air bubbles created during the preliminary bonding in the interface of the glass slides. To investigate the effect of these cavities on the prevention of bubbles, images from a digital microscope (Hirox KH-7700, Hackensack, NJ) of the glass/SU-8 interfaces were taken for microdevices (SU-8 cured) with and without such structures.

### Uniformity and roughness of the SU-8 film

The uniformity of the SU-8 films deposited by spinning on glass was evaluated by considering two options: **A**, single-step and **B**, double-step rotation. For **A**, the deposition was taken at 3000 rpm for 30 s. For **B**, two sequential steps were used: 500 rpm for 5 s (spread cycle) and 3000 rpm for 25 s (spin cycle) with the same total time of 30 s. The steps of pre-bake, UV exposure, and PEB were conducted as described earlier. Uniformity of these films was assessed by measuring the thicknesses at seven points along each slide by profilometry. Ten glass slides were analysed for each applied protocol. Atomic force microscopy (AFM, Veeco MultiModeTM SPM equipment, Plainview, NJ) measured the RMS of the film resulting from process **B**.

### Cross-sectional view of the microchannel

Field-emission gun scanning electron microscopy (SEM-FEG) by a Philips XL 30 (Eindhoven, Netherlands) and FEI Inspect F50 (Hillsboro, OR) produced images for analysing the cross-section of the bonded microchannel. To improve the image contrast, samples were metalized by employing a BAL-TEC SCD 050 (Balzers, Liechtenstein). Micrographies were performed for two methods of development: pumping the developer (1) before SU-8 cure under specific time (10 s) and flow rate (5 μL min^−1^) and (2) after selective cure of SU-8 by using Al film in the microchannel as absorption standard during UV exposure (Fig. 1).

### Adhesion strength

The adhesion strength was evaluated by two methods: leakage pressure and tensile pull tests. In the first case, the pressure inside the channel produced by water pumping at different flow rates was measured in three microchips. Pump of high performance liquid chromatography (HPLC, Shimadzu LC-10AVP, Columbia, MD) was used to carry water and measure the pressure. The difference between the chips was relative to processing stage of the SU-8 film: (1) post pre-bake (uncured), (2) post UV exposure (with produced cure initiator but uncured), and (3) post PEB (cured). For the tensile pull tests, we used EMIC DL-3000 equipment (São Paulo, Brazil) with a speed of 10^−3^ mm min^−1^. The bonding strength was determined by the ratio between the breaking force and the area of steel pieces (124.69 mm^2^). These pieces were connected to the equipment charge cell and glued to both sides of the chips by Torr Seal^®^ (Agilent Technologies, Santa Clara, CA).

### Effect of the remaining SU-8 on channel property

The fabrication of glass microchannels by the SAB method is limited by one condition: the effect of the residual SU-8 layer on properties of the surface inside the microfluidic channel should be negligible. Aiming to validate the SAB for bonding of glass microdevices, we studied this effect by evaluating the capacity of heat dissipation in electrophoretic applications employing the glass/SU-8/glass microchannels obtained by SAB. The data were compared with those observed recently in our group for glass and glass/SU-8 (without the SU-8 development) chips^25^. All of the microchannels were carved as previously mentioned. The microfluidic channels consisted of a 27-mm-long straight channel with reservoirs in both ends. Micropipette tips were used as reservoir supports for the buffer solutions. These were fixed by epoxy glue on the substrate. Glass chips were thermally bonded in a muffle furnace (EDG F3000) at 590 °C for 480 min under the application of 1.6 kg weight with a progressive temperature ramp of 2 °C min^−1^. Glass/SU-8 devices were fabricated as previously cited in the literature^26^. The bonding involved neither selective development of the SU-8 adhesive nor deposition of Al inside the microchannel, as proposed here.

Heat dissipation was investigated through Ohm’s law plots by measuring the relationship among current and applied voltage. A bipolar single-channel high-voltage power supply (CZE 1000R, Spellman, Hauppauge, NY) was employed. Additionally, a computer with a National Instruments interface (USB-6009 model) was used to control the power supply whereas the data acquisition was performed with software written in LabVIEW^®^. Microchannels were rinsed with 100 mmol L^−1^ NaOH for 30 min prior to any measurement to create negatively charged groups on walls. Afterwards, cleaning of the microchannels with deionized water and 20 mmol L^−1^ phosphate buffer at pH 8.0 was performed for 20 and 10 min, respectively. The buffer was intended to equilibrate the electric double layer and pH on the walls of the microchannels. Lastly, all of the reservoirs were filled with 100 μL phosphate solutions. The preconditioning of the surface was done by applying a vacuum in the waste reservoir. NaOH was used to adjust the buffer pH for 8.

## Additional Information

**How to cite this article**: Lima, R. S. *et al.* Sacrificial adhesive bonding: a powerful method for fabrication of glass microchips. *Sci. Rep.* **5**, 13276; doi: 10.1038/srep13276 (2015).

## Supplementary Material

Supplementary Information

Supplementary Video

## Figures and Tables

**Table 1:** Comparison of the alternative methods to the thermal direct technique for glass microchip bonding.

Bonding Method	Time	Cost	Viability for ULSI	Adhesion Strength	Reference
Low temperature after long period of contact among the slides	High	Moderate	No	High	46
Low temperature after surface activation in liquid phase	Moderate	Moderate	No	Moderate	47
Low temperature after surface activation in liquid phase	Moderate	Low	No	High	48
Low temperature after surface activation in liquid phase	High	Low	No	Moderate	49
Low temperature after surface activation in gas phase	High	Low	No	Moderate	50
Low temperature after functionalization with silanol/amine groups	High	Moderate	No	High	51
Room temperature after surface activation in argon plasma	Low	High	Yes	High	52
Room temperature after surface activation in O_2_/N_2_ plasma	Low	High	Yes	High	53
Room temperature after surface activation in O_2_/CF_4_ plasma	Moderate	High	Yes	High	54
Room temperature after surface activation in liquid phase	High	Low	No	High	55
Sacrificial adhesive bonding (SAB)	Low	Low-Moderate	Yes	High	This paper
