# Supplementary material for: Sacrificial adhesive bonding: a powerful method for fabrication of glass microchips
Source: Sci Rep. 2015 Aug 21;5:13276. doi: 10.1038/srep13276 (PMC4543966; doi:10.1038/srep13276)
Supplement: Supplementary Information [file srep13276-s1.pdf]

# Sacrificial adhesive bonding: a powerful method for fabrication of glass microchips

Renato S. Lima,[1,2] Paulo A. G. C. Leão,[3,4] Maria H. O. Piazzetta,[1] Alessandra M. Monteiro,[3,4] Leandro Y. Shiroma,[1,2] Angelo L. Gobbi,[1] and Emanuel Carrilho*[3,4]

[1] Laboratório de Microfabricação, Laboratório Nacional de Nanotecnologia, Centro Nacional de Pesquisa em Energia e Materiais, Campinas, São Paulo 13083-970, Brasil.
[2] Instituto de Química, Universidade Estadual de Campinas, Campinas, São Paulo 13083-970, Brasil.
[3] Grupo de Bioanalítica, Microfabricação e Separações, Universidade de São Paulo, São Carlos, São Paulo 13566-590, Brasil. Tel.: 16 3373 9441.
[4] Instituto Nacional de Ciência e Tecnologia em Bioanalítica, Campinas, São Paulo 13083-970, Brasil.

# Supplementary Information

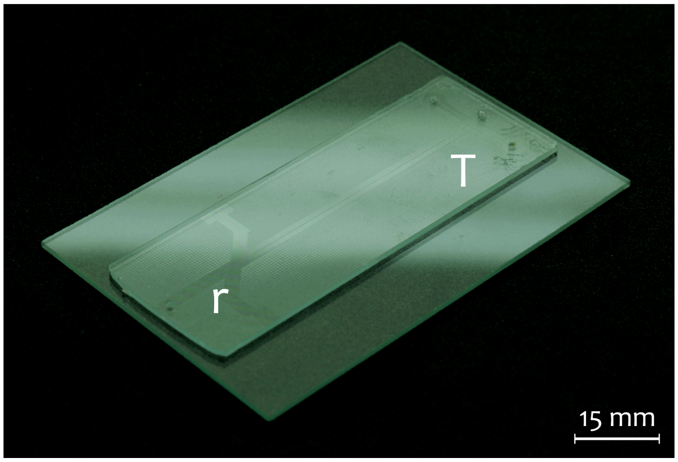


**Figure S1.** Photo of a glass microdevice obtained by the pressure-assisted bonding above the SU-8 glass transition temperature reported in this article. Such procedure aimed only to show the bonding of long and wide microfluidic channels by using the experimental protocol adopted herein. Thus, the SU-8 selective development was not made. In this case, the microchannels presented 65 and 15 mm long and 140 µm width. We can visualize in this photo the air-trapping zones carved in the top slide (T) around the channels and the region (r) close to the end of the microchannel, which was sheltered to any steps of electrode deposition.

## Video: "Adhesion Strength.mp4"

Tensile pull test for one sample that fractured over a pressure of 7.04 MPa
